# Transcriptome, physiological and biochemical analysis of *Triarrhena sacchariflora* in response to flooding stress

**DOI:** 10.1186/s12863-019-0790-4

**Published:** 2019-11-29

**Authors:** Jia Wang, Han Sun, Jiajin Sheng, Surong Jin, Fasong Zhou, Zhongli Hu, Ying Diao

**Affiliations:** 1 State Key Laboratory of Hybrid Rice, Hubei Lotus Engineering Center, College of Life Sciences, Wuhan University, Wuhan, 430072 People’s Republic of China; 2 Institute of Special Plants, College of Landscape Architecture and Life Science, Chongqing University of Arts and Sciences, Chongqing, 402160 People’s Republic of China; 3 School of Chemistry, Chemical Engineering and Life Science, Wuhan University of Technology, Wuhan, 430070 People’s Republic of China; 4 College of Life Sciences, Nantong University, Nantong, 226019 People’s Republic of China

**Keywords:** Flooding stress, Physiological, Biochemical, Nakai, Transcriptomes

## Abstract

**Background:**

In recent decades, the frequency of flooding is increasing with the change of global climate. Flooding has become one of the major abiotic stresses that seriously affect growth and development of plants. *Triarrhena sacchariflora* Nakai has been considered a promising energy crop for utilization in ethanol production. Flooding stress is among the most severe abiotic stressors in the production of Nakai. However, the physiological and molecular biological mechanisms of Nakai response to flooding is still unclear. In the present study, in order to understand the molecular mechanisms of Nakai in response to flooding stress, the transcriptome, physiological and biochemical were investigated.

**Results:**

The results demonstrated that significant physiological changes were observed in photosynthetic system, antioxidative enzyme activity, chlorophyll, carotenoid, proline, lipid peroxidation and soluble sugar content under normal and flooding treatments. Such as, the chlorophyll, carotenoid contents and photosynthetic system were significantly decreased. Whereas, the antioxidative enzyme activity, proline, lipid peroxidation and soluble sugar has increased first and then decreased under treatments compared with the normal plants. Additionally, a total of 8832, 6608 and 3649 unigenes were validated to be differentially expressed under different treatments, respectively. Besides, gene ontology enrichment and Kyoto Encyclopedia of Genes and Genomes pathway enrichment analysis of the different expression levels of genes also presented processes, which involved in photosynthesis, sucrose catabolism, glycolysis, stress response and defense, phytohormone biosynthesis and signal transduction.

**Conclusions:**

The results provide a comprehensive view of the complex molecular events involved in the response to flooding stress of Nakai leaves, which also will promote the research in the development of flood-resistant crops and provide new tools for Nakai breeders.

## Background

*Triarrhena sacchariflora* (Maxim.) Nakai is a large perennial herbaceous plant, belonging to the Miscanthus spp. of Gramineae, which is widespread in the northern and midland of China [1, 2]. Nakai is an important plant resource with great potential because of its high biomass yield, broad adaptability, strong regeneration capacity and great potential for carbon sequestration. Thus, it has been considered a promising energy crop for utilization in ethanol production [1, 3]. Meanwhile, Nakai as a promising energy plant which can be planted in marginal and semi-marginal lands, and not competing for lands use for food crops. It is a C4 plant with high photosynthetic efficiency and high caloric value of dry matter. Besides, Nakai has been widely applied to soil and water conservation, pulp and paper, landscape gardening and medicine [4, 5]. Therefore, Nakai not only has low inputs cost in fertilizer, pesticide and soil management, but also has highly tolerance to heavy metals and saline soils. Previous studies have indicated that Nakai can tolerate high concentrations of heavy metals by reducing, accumulating and compartmentalization, and play an important role in wetland ecosystems [4, 6].

With the change of global climate, the frequency of flooding is increasing. Flooding has become one of the major abiotic stresses that seriously affect growth and development of plants [3, 7]. The excess water limits some certain basic resources in the environment for plants, such as oxygen and carbon dioxide [8–10]. In short, flooding condition imposes serious threat to plant survival by disturbing normal growth and development, hampering different physiological and metabolic activities, which includes reduction in stomatal conductance, CO2 assimilation rate, photosynthesis rate, and nutritional imbalance. These adverse effects might lead to loss of crop yield, and severe suffering may result plant death [7, 11–13]. Fortunately, plant has developed different morphological, anatomical, and physiological adaptive features to adapt the flooding stress, for instance, formation of adventitious roots and aerenchyma, enhances the production of ethylene, and increases ADH activities [11, 14]. Meanwhile, with the oxygen limiting, anaerobic respiration starts to become the primary pathway for supplying energy to the plants [9, 11, 15]. Such as, glycolysis process, ethanolic fermentation, pyruvate metabolism and alanine metabolism were elevated to increase substrate-level production of ATP [11, 16, 17]. The limited oxygen also triggers the accumulation of reactive oxygen species (ROS), resulting in membrane lipid peroxidation, protein and nucleic acid structure changes, and low antioxidant enzyme activity [10, 18, 19]. ROS-scavenging enzymes, such as superoxide dismutase (SOD), catalase (CAT), and peroxidase (POD**)**, are changed under flooding stress due to the disturbed balance of ROS generation and removal [20–23]. Higher cellular levels of ROS damage plants through oxidation of different cell components, and the degree of damage is estimated through the accumulation of malondialdehyde (MDA) which is the final product of membrane lipid peroxidation. Besides, previous studies indicated that increase in soluble sugars and proline can offer protection for flooded plants by improving cell osmotic potential [24, 25].

In recent decades, the physiological and molecular mechanisms of adaptation to flooding stress in many plants have been investigated [7, 9, 11, 13]. Some studies have identified that flood-tolerant plants generally are specific survival strategies including the low-O2 escape syndrome (LOES) and low-O2 quiescence syndrome (LOQS) are extremes [26]. These processes are mainly driven by phytohormones, including ethylene, Auxin (IAA), gibberellin (GA), abscisic acid (ABA), cytokinine (CK), and salicylic acid (JA) [27–31]. Besides, the VII ethylene response factor transcription factors (TFs) are involved in the activation for anaerobic metabolism in *Arabidopsis thaliana* [32]. However, compared to other crops, the molecular mechanism of flood adaptation in Nakai still remains unclear, which may reflecting the limited availability of genomic and transcriptional resources. Recently, with the development of next-generation sequencing technologies, RNA-Seq has been used to elucidate plant responses to various biotic and abiotic stresses, including floods and droughts [7, 33–36]. Such as in *Arabidopsis thaliana* [36, 37], rice [38], soybean [39, 40], maize [41], canola [33], Legume [42] and so on. These studies have identified a large number of environmental stress genes, which involved in the processes of energy metabolism and photosynthesis, carbohydrate mobilization and sucrose catabolism, stress response and defense, phytohormone biosynthesis and signal transduction.

In the present study, we analyzed the physiological and transcriptome responses to different levels of flood treatment in *Triarrhena sacchariflora* (Maxim.) Nakai leaves. Moreover, we examined the accumulation and change of photosynthetic rates, photosynthetic pigments, proline, antioxidative enzyme activity, lipid peroxidation (MDA) and soluble sugars. The results described the changes of physiological, biochemical and the complex molecular mechanisms in response to flooding stress at different treatment times, which is helpful to our understanding of plants response to flooding stress.

Meanwhile, these transcriptome databases also will promote the research in the development of flood-resistant crops and provide new tools for Nakai breeders.

## Results

### Chlorophyll and carotenoid content

To dissect the influence of Chlorophyll and carotenoid contents under different times of flooding stress, we determined the chlorophyll a, b, total and carotenoid content of normal (0-DAF) and after samples treatment for 2, 4, 6 and 8 days after flooding (DAF). The results showed that the chlorophyll a, b, total and carotenoid content were significantly decreased with the continuation of flooding treatment (Table 1). In addition, the ratio of chlorophyll a/chlorophyll b were gradually larger under flooding conditions.

**Table 1:** The Chlorophyll and carotenoid contents effect of leaves by flooding stress

Time (DAF)	Chlorophylla (μgg-1 FM)	Chlorophyll b (μgg-1 FM)	Ratio of a/b	Chlorophyll (μgg^− 1^ FM)	Carotenoids (μgg^− 1^ FM)
0	1012.2 ± 56.1 a	628.4 ± 26.1 a	1.61 ± 0.1a	1640.58 ± 101.5 a	215.2 ± 13.2 a
2	946.4 ± 29.2 b	463.4 ± 19.8 b	2.04 ± 0.1b	1409.8 ± 105.7 b	162.6 ± 16.1 b
4	917.5 ± 26.3 bc	427.9 ± 22.3 c	2.14 ± 0.1c	1345.35 ± 112.2 b	152.8 ± 21.4 b
6	885.1 ± 25.8 cd	338.5 ± 11.8 d	2.61 ± 0.1d	1223.6 ± 32.4 c	145.8 ± 18.4 b
8	861.1 ± 23.1 d	336.1 ± 21.1 d	2.56 ± 0.1d	1197.2 ± 24.8 c	98.6 ± 8.4 c

Vertical bars represent the mean standard error (mean ± SE; n = 6). Means followed by different letters indicate significant differences at *p* < 0.05 according to LSD test

### Gas exchange attributes

Leaf gas exchange in Naka was measured for 0, 2, 4, 6 and 8 DAF, starting at the onset of flooding treatment (Table 2). With increasing duration of flooding, assimilation rates completely inhibited, which 58.3% decrease in 8 DAF. The flooding stress caused increase of stomatal conductance and transpiration rate in 2-DAF. But, from the fourth day to the end of the experiment, stomatal conductance and transpiration rate were decreased significantly with the exposure of flooding stress. Water use efficiency was lower in flooding treatment plants in contrast to normal control plants. On the contrary, the internal CO2 was increased with the exposure of plants to the flooding stress, which is increased 3.5-fold by 8-DAF. Thus, the flooding stress significantly decreased the photosynthetic activity of leaves, and the level of influence increased with the time of exposure to flooding stress.

**Table 2:** The effects of flooding stress on photosynthetic parameters of the leaf in Naka

Time (DAF)	Internal CO2 (ppm)	Stomatal Conductance (mmol m-2 s-1)	Assimilation Rate (umol-2 s-1)	Transportation Rate (m-2 s-1)	Water Use Efficiency (%)
0	31.67 ± 1.9 a	54.33 ± 3.1 d	11.5 ± 0.5 c	1.83 ± 0.06 b	6.29 ± 0.6 c
2	63 ± 1.3 b	58.5 ± 2.9 e	10.7 ± 0.3 c	2.26 ± 0.07 c	4.69 ± 0.4 a
4	88 ± 2.1 c	38.78 ± 1.8 c	7.15 ± 0.4 b	1.33 ± 0.08 a	5.38 ± 0.7 b
6	108 ± 9.2 d	34 ± 1.1 b	5.75 ± 0.6 ab	1.26 ± 0.06 a	4.58 ± 0.3 a
8	111 ± 8.3 d	28 ± 1.0 a	4.8 ± 0.4 a	1.05 ± 0.04 a	4.57 ± 0.3 a

Vertical bars represent the mean standard error (mean ± SE; n = 6). Means followed by different letters indicate significant differences at *p* < 0.05 according to LSD test

### Antioxidant enzyme activities

**Fig. 1:**
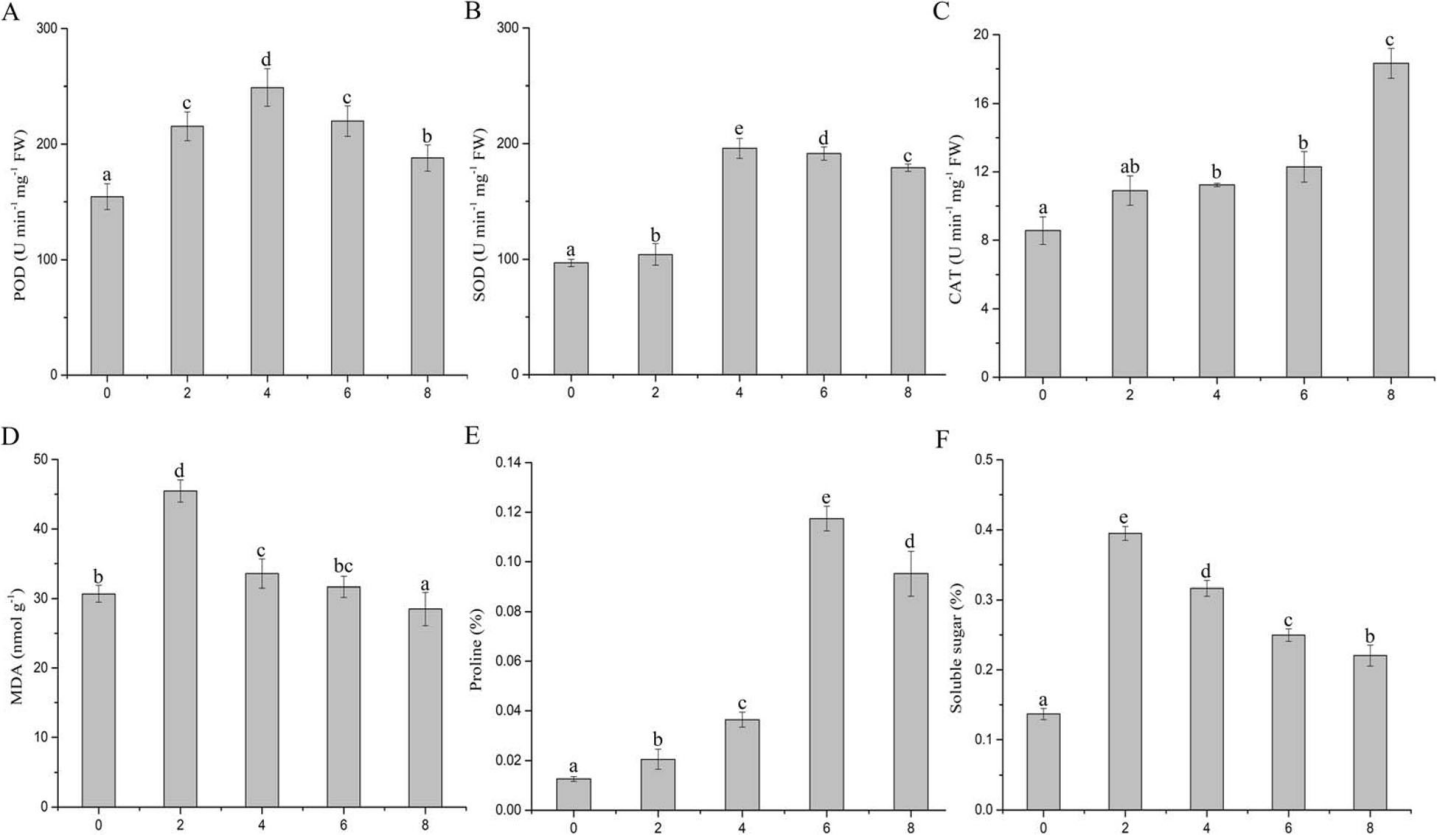
Leaf POD, SOD, CAT, MDA, proline and soluble sugar content of Nakai under control and flooding conditions. Vertical bars represent the mean standard error (mean ± SE; *n* = 6). Means followed by different letters indicate significant differences at *p* < 0.05 according to LSD test

To investigate the activity of the antioxidative enzymes in Nakai leaves subjected to different times of flooding stress, the catalase (CAT), superoxide dismutase (SOD) and total peroxidase (POD) were evaluated (Fig. 1a-c). Accordingly, there was a significantly increase in the activity of CAT in leaves of plants exposed to flooding compared to normal plants with increasing duration of flooding. Maximum activity of CAT occurred in the 8-DAF treatments. Besides, the activity of SOD and POD were followed the same trend, which has increased first and then decreased under treatment plants and compared with the control plants. Maximum activity of SOD and POD occurred in the 4-DAF treatments, 195.9 ± 11.2 U min^− 1^ mg^− 1^ FW and 249 ± 14 U min^− 1^ g^− 1^ FW, respectively. After that, the activity of enzymes content reduced relative to the level but was still higher than the control plants. Thus, our results suggest that the Nakai increases the activity of the antioxidative enzymes to adapt the flooding stress, which could keep intracellular oxidative homeostasis.

### Lipid peroxidation, free proline and soluble sugar content

Lipid peroxidation is used as an indicator of oxidative stress. Free radical formation and membrane damage levels were analyzed by measuring the MDA content in the plants during flooding stress. The results showed that the MDA content has increased first and then decreased under treatment plants compared with the normal plants (Fig. 1d)**.** Lipid peroxidation levels increased to the maximum at 2-DAF. The content of Proline also changed similarly to that of MDA. They increased at first and then decreased significantly when the periodical flooding time was longer than 6 days (Fig. 1e-f). The maximum proline was observed in the 6 days treatment at 0.12 ± 0.01%. The level of sugar content was increased in treatment plants when compared to control plants (Fig. 1f). The sugar content reached the maximum on the second day of water flooding. After that, the content reduced relative to the level but was still higher than the control.

### Illumina sequencing, assembly and gene annotation

**Fig. 2:**
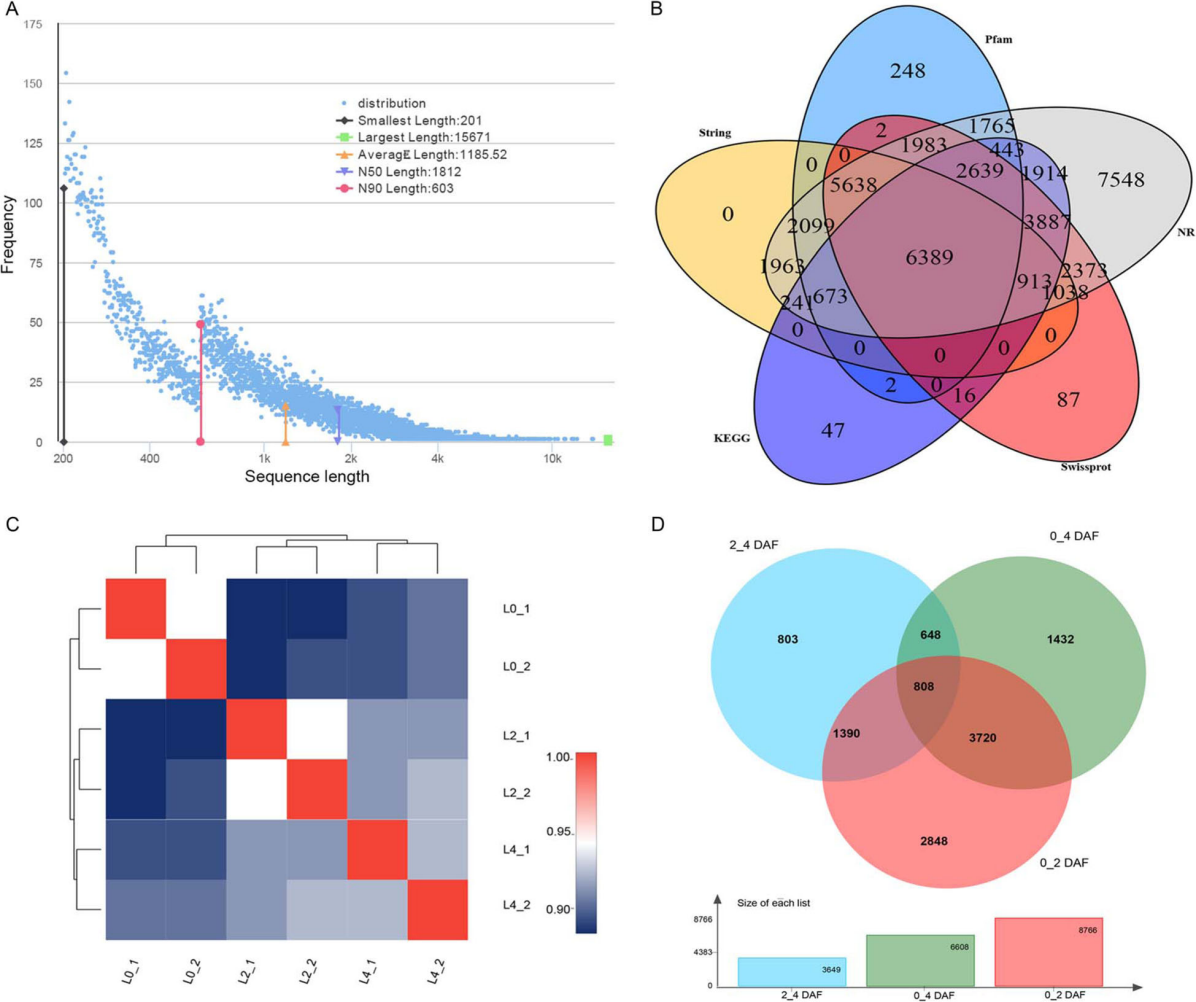
**a-b** the sequence length distribution, (**c**) Venn diagrams of the unigenes annotation in different public protein database, (**d**) Venn diagrams of differentially expressed genes (DEGs) in response to flooding

Based on the previous results, we found that the changes in these physiological and biochemical were mainly concentrated in the first three treatments (0-DAF, 2-DAF, 4-DAF). Therefore, to further exploration the physiological responses and the transcriptome information profiles of the three treatments in Nakai, we performed a set of consecutive and comprehensive transcriptome sequencing from the 3 treatments samples of flooding stress. A total of 102,590,796, 111,241,228 and 108,426,642 clean reads were obtained from the L0- DAF, L2-DAF and L4-DAF samples, respectively (Additional file 7: Table S1). The strategy of pooling all clean reads together generated, 58,908 unigenes were obtained with a N50 length of 1812 bp after assembly (Fig. 2a-b). Besides, to validate and annotate the assembled unigenes, all unigenes were aligned against public protein database using BlastX with a cut-off of value<1e-5. In total 41,908 (71.14%) unigenes were annotated, with 41,507 (70.46%) unigenes in NR, 24,966 (42.38%) unigenes in Swissprot, 17,165 (29.14%) unigenes in KEGG, 18,955 (32.18%) unigenes in String and 21,882 (37.15%) unigenes in Pfam database (Fig. 2c and Additional file 8: Table S2). Furthermore, 17,000 (28.86%) unigenes did not show significant match sequences from these databases, likely representing unknown genes, and 6389 (10.85%) unigenes were annotated in all databases.

### Identification and analysis of differentially expressed genes for flooding stress

In order to test whether the variation of biological duplication between samples accords with the expectation of experimental design, samples correlation and PCA analysis were adopted. The PCA analysis and hierarchical clustering revealed that the six samples clustered in three corresponding discrete group, which can used to the requirements of subsequent experiments (Additional file 1: Figure S1).

To investigate the differentially expression levels of genes (DEGs) between the normal and treatment samples, all assembled unigenes were analyzed using the edgeR software, with an FDR < 0.05 and |logFC| ≥ 1 as a cutoff. A total of 8766 (0_2-DAF: 0-DAF vs 2-DAF) and 6608 (0_4DAF: 0-DAF vs 4-DAF) genes were identified as being differentially expressed at normal samples, relative to treatment samples, respectively (Fig. 2d and Additional file 9-10: Table S3-S4). These DGEs may have important function implication in flooding tress for Nakai. On the other hand, 3649 (2_4-DAF: 2-DAF vs 4-DAF) genes were revealed to be significantly differentially expressed between two treatment samples, which indicate there has a difference between treatment samples under flooding tress. Besides, the number of DEGs in 0_2-DAF was remarkably higher than other pairwise comparisons, indicating the involvement of important complex developmental procedures in early stage of flooding stress. Moreover, Venn diagram depicted the overlapped expressed DEGs and specifically expressed DEGs in 0_2-DAF, 0_4-DAF and 2_4-DAF (Fig. 2d). Among these DEGs, 808 were common to both group, 2848 unique to 0_2-DAF, 1432 unique to 0_4DAF and 803 unique to 2_4-DAF, respectively. On the other hand, to verify the reliability of the RNA-seq data, eight DEGs were randomly selected for further investigation using qRT-PCR methods. The qRT-PCR results of these DEGs were highly consistent with the RNA-seq data (Table 3), which confirming the RNA-seq data are reliable.

**Table 3:** Quantitative real-time PCR (qRT-PCR) validation and RNA-seq data of eight selected DEGs in Nakai

Unigene ID	Description	Log2|FC|(L2/L)		Log2|FC|(L4/L)		regulate
		RNA-Seq	qRT-PCR	RNA-Seq	qRT-PCR	
TRINITY_DN27789_c0_g2	Ethylene-responsive transcription factor 1B	10.28	11.13 ± 1.02	11.24	10.81 ± 0.82	Up
TRINITY_DN13863_c0_g1	Auxin-responsive protein SAUR41	8.32	6.53 ± 0.61	9.34	10.94 ± 0.57	Up
TRINITY_DN28460_c2_g3	AP2/ERF and B3 domain-containing protein	7.95	4.25 ± 0.12	8.03	6.37 ± 0.29	Up
TRINITY_DN25549_c0_g2	Hexokinase-8	7.16	6.93 ± 0.31	8.06	8.32 ± 0.12	Up
TRINITY_DN26663_c1_g3	peroxidase activity, Peroxidase 12	6.68	5.38 ± 0.27	7.26	6.43 ± 0.14	Up
TRINITY_DN33654_c2_g7	Chlorophyll A-B binding protein	5.07	6.37 ± 0.11	2.3	2.15 ± 0.07	Down
TRINITY_DN28966_c0_g2	Jasmonate ZIM domain-containing protein 10	6.1	3.88 ± 0.09	4.3	2.18 ± 0.06	Down
TRINITY_DN30453_c1_g5	Nitrate reductase	7.09	7.32 ± 0.30	4.7	4.53 ± 0.0.12	Down

In order to classify the functions of the DEGs of Nakai, these DEGs were assigned based on Gene ontology (GO) analysis. The results were shown in Additional file 2: Figure S2. The number of the DEGs in each category for GO annotation showed parallel proportion between 0_2-DAF and 0_4-DAF (Additional file 2: Figure S2a-b). The up-regulated and down-regulated DEGs could be classified into 51 and 59 GO categories respectively. Among these categories, metabolic process, binding, cellular process and catalytic activity were the four dominant groups. But, the results are different compared with of 2_4-DAF, which has 54 and 45 categories of up-regulated and down-regulated DEGs, respectively (Additional file 2: Figure S2c).

### GO functional enrichment and KEGG pathway enrichment analysis of DEGs

**Fig. 3:**
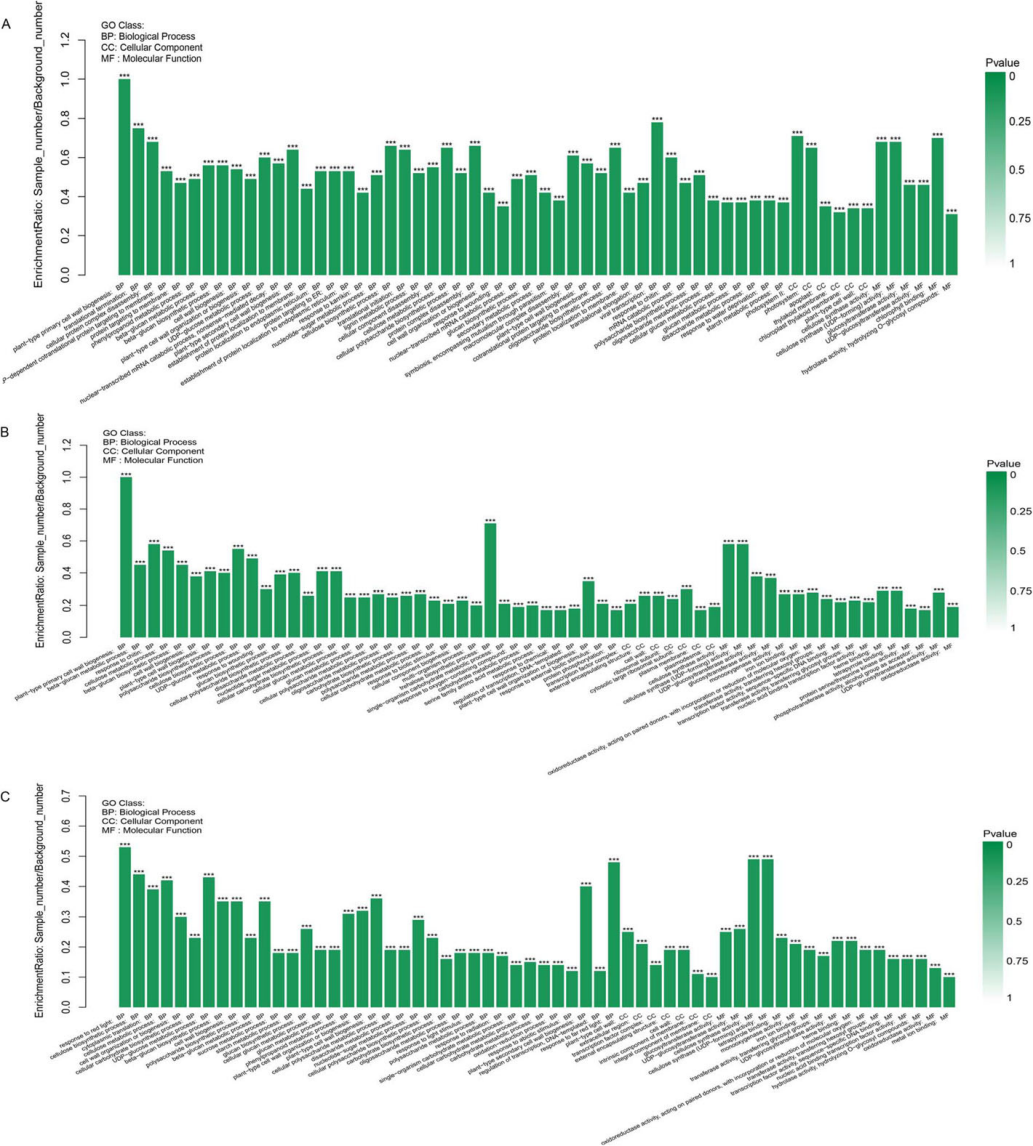
Gene ontology (GO) functional enrichment analysis of DEGs between normal and treatment samples. **a** Most enriched GO terms in 0_2-DAF, (**b**) Most enriched GO terms in 0_4-DAF, (**c**) Most enriched GO terms in 0_4-DAF. The height of the column, the ordinate said enrichment rate. Color denotes the significance of enrichment, namely *P*value. The mark of *P*value< 0.001 is ***, the mark of *P*value< 0.01 is **, the mark of *P*value< 0.05 is *, and the color gradient on the right is the size of *P*value

To further evaluate these DEGs of Nakai, we considered the biological function of the DEGs that were regulated in response to flooding. These DEGs of 0_2-DAF, 0_4-DAF and 2_4-DAF were performed to enrichment analysis based on GO and KEGG pathways. GO enrichment analysis of DEGs indicated that 324, 182 and 143 GO terms were significantly enriched with the criteria of FDR < 0.05, respectively (Fig. 3 and Additional file 11: Table S5). Moreover, 87 GO terms were in all three points of 0_2-DAF, 0_4-DAF and 2_4-DAF. Of them, 54 were enriched in the category of biological process (e.g., response to oxidative stress, response to abiotic stimulus, plant-type primary cell wall biogenesis), 23 in molecular function (e.g., peroxidase activity, monooxygenase activity, cellulose synthase activity), and 10 in cellular component (e.g., photosystem, cell wall, membrane part). By 0_2-DAF, 155 unique GO terms were enriched including defense response, response to water, secondary metabolic process and photosystem II. In contrast, the 10 unique significantly enriched terms were specific to the 0_4-DAF (e.g., malate dehydrogenase (decarboxylation) (NAD+) activity, regulation of gene expression, cytosolic large ribosomal subunit). These differences in enriched GO terms between normal and treatment samples, which indicate these terms could be differentially implicated in response to flooding stress in Nakai.

**Fig. 4:**
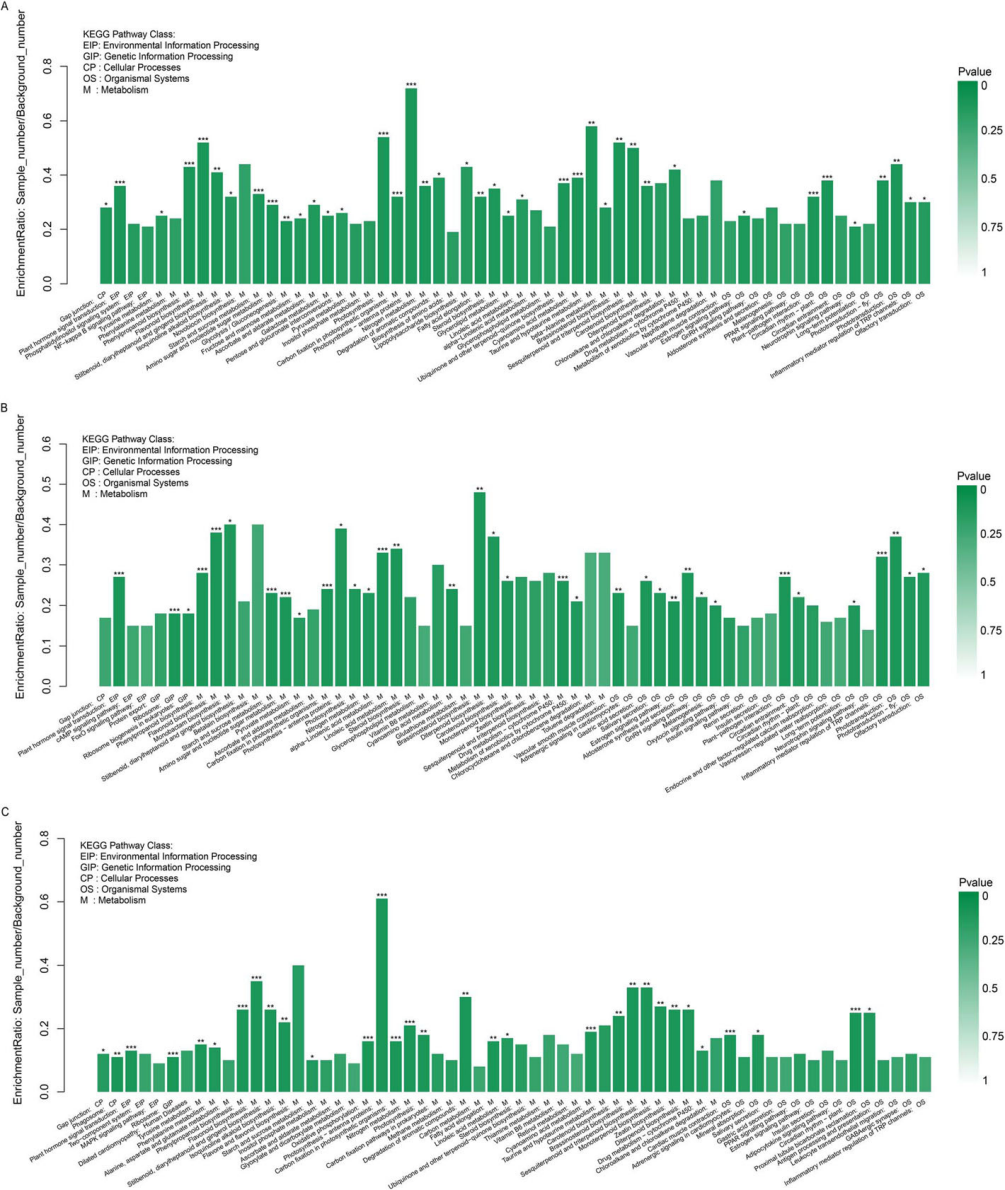
Significantly enriched KEGG pathways of DEGs functions. **a** Most enriched pathways in 0_2-DAF, (**b**) Most enriched pathways in 0_4-DAF, (**c**) Most enriched pathways in 2_4-DAF. The height of the column, the ordinate said enrichment rate. Color denotes the significance of enrichment, namely *P*value. The mark of *P*value< 0.001 is ***, the mark of *P*value< 0.01 is **, the mark of *P*value< 0.05 is *, and the color gradient on the right is the size of *P*value

On the other hand, these DEGs were matched in the KEGG database and assigned to 375 pathways (Additional file 8: Table S2). Among these categories, metabolic pathways, biosynthesis of secondary metabolites, biosynthesis of antibiotics and microbial metabolism in diverse environment were the four dominant pathways. Besides, with the KEGG enrichment pathways analysis (*p*-value< 0.05), 34, 35 and 30 KEGG pathways were significantly enriched between the normal and treatment samples, respectively (Fig. 4 and Additional file 12: Table S6). Among these pathways, 13 enriched pathways were common to all three groups. The most significant enrichment pathways were phenylpropanoid biosynthesis pathway, plant hormone signal transduction, Starch and sucrose metabolism and photosynthesis, followed by pathways of carbon fixation in photosynthetic organisms, nitrogen metabolism and photosynthesis - antenna protein. Besides, a number of DEGs were enriched in 10 unique pathways to 0_2-DAF (e.g., Ascorbate and aldarate metabolism, Glycolysis / Gluconeogenesis, Galactose metabolism), whereas17 unique pathways to 0_4-DAF (e.g., Diterpenoid biosynthesis, Phototransduction, Ribosome biogenesis in eukaryotes) and 8 unique pathways to 2_4-DAF (e.g., Oxidative phosphorylation, Zeatin biosynthesis, Phenylalanine metabolism). Enriched GO terms and enriched KEGG pathways analysis could provide a probable insight into the molecular mechanism of flooding stress response.

### Flooding lead to changes in hormonal responses and biosynthesis-related DEGs

**Fig. 5:**
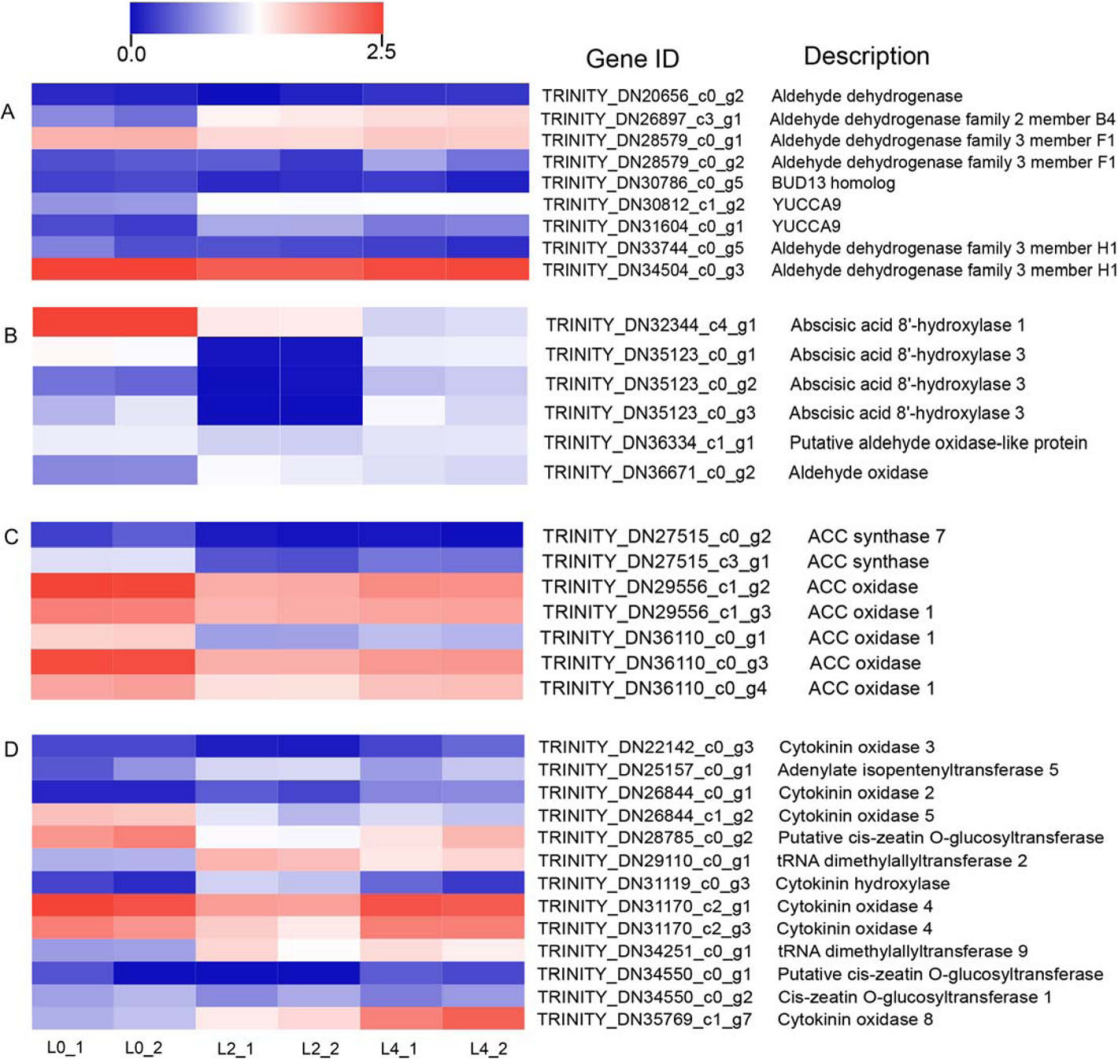
Heatmap analysis of DEGs involved hormone response and biosynthesis of IAA, ABA, ethylene and cytokinin. The expressions of gene are displayed different colors. Red means high expression, and blue means low expression

In this study, a total of 108 DEGs were identified that related to plant hormone signal transduction, including 32 auxin (IAA), 21jasmonic acid (JA),18 abscisic acid (ABA), 9 cytokinine (CK), 7 ethylene, 7salicylic acid (SA), 7 brassinosteroid (BR), 4 gibberellin (GA) genes (Additional file 3: Figure S3). On the other hand, IAA metabolism was observed with 9 DEGs identified during flooding stress, including 6 Aldehyde dehydrogenase, two indole-3-pyruvate monooxygenase (YUCCA) and a BUD13 homolog genes (Fig. 5a). A total of 6 associated with ABA metabolism were significantly altered during flooding, including 4 ABA 8′-hydroxylase and two aldehyde oxidase genes (Fig. 5b). The aldehyde oxidase was significantly induced during flooding stress and the ABA 8′-hydroxylase was suppressed, which can promote the accumulation of ABA. In addition to IAA and ABA associated DEGs, DEGs associated with the ethylene (ET) metabolism were also observed. These included two ACC synthetase and five ACC oxidase genes, most of which showed the greatest changing during flooding stress (Fig. 5c). Besides, CK metabolism was observed with 13 DEGs identified across the time course. These genes included three cytokinin oxidase, two tRNA dimethylallyltransferase, one cytokinin hydroxylase and one adenylate isopentenyltransferase genes, all of which were induced by flooding stress (Fig. 5d). In contrast, three Cis-zeatin O-glucosyltransferaseand and three cytokinin oxidase were downregulated in treatment plants.

### Identification and analysis of TFs

In this study, A total of 559 putative TFs were identified using a local Blastp (e-value <1e-8) homology search against the PlantTFDB. These TFs were classified into 32 TF families in this dataset (Additional file 13: Table S7). The most abundant TF family was MYB proteins, followed by AP2/ERF, bHLH, GRAS, NAC, C2C2, bZIP and WRKY protein families. To provide insights into the regulatory of TFs for flooding stress, we examined these DEGs of TFs in different samples. Totally, 205 (169 in 0_2-DAF, 132 in 0_4-DAF, 86 in 2_4-DAF) TFs were observed with 123 up-regulated and 82 down-regulated TFs belonging to 31 different families (Additional file 4: Figure S4 and Additional file 10: Table S4). Furthermore, the most enriched TF families in the up-regulated DEGs were MYB proteins, followed by AP2/ERF, NAC, GRAS and bZIP protein families; whereas the most abundant TF family in the down-regulated DEGs was AP2/ERF protein, followed by MYB, NAC, WRKY and bHLH proteins.

## Discussion

With the continuous development of global warming, the frequency of floods is increasing. Floods stress has seriously affected the plant growth, development and yield. Fortunately, plants have evolved some abilities to resist flood habitats by escaping phenotypes during long-term adaptive evolution. Such as, numerous studies have demonstrated that a variety of physiological, biochemical and molecular changes have been characterized in plants subjected to flooding conditions. These changes mainly include stomatal closure, forming adventitious roots and aerenchyma, dormancy and fast elongation by leaves accompanied [7, 11, 12, 43]. The RNA-Seq technology is widely used for transcription profiling of plants growing under different stresses. So far as we know, there is no report for the transcriptome responses of flooding stresses in Nakai. Thus, to further dissect the effects of different levels of flooding, we investigated the physiological and molecular responses (transcriptomics) of under different levels of flooding treatments in Nakai, which provides straight forward information regarding the response of flooding stresses.

In the present study, a total, 58,908 unigenes were obtained with a N50 length of 1812 bp after assembly. Thousands of DEGs have been identified in Nakai leaves in response to flooding stress. These DEGs are mainly involved in photosynthesis, plant hormone signal transduction, phenylpropanoid biosynthesis and cellulose biosynthetic process, Starch and sucrose metabolism, stress response and defense, Nitrogen metabolism, oxidation-reduction process. The similar results have been reported by other studies, which indicate that these reactions play vital roles in response of Nakai to flooding.

### Photosynthetic system response to flooding

Flooding usually inhibited the rate of photosynthesis in plants. And this inhibition is positively correlated with the advancing of flooding duration. In the present study, the physiological experiment demonstrated that the chlorophyll a, b, total carotenoid contents were significantly decreased under flooding conditions, which is consistent with the transcriptome reported. Two DEGs were detected from the porphyrin and chlorophyll pathway (Ko00860). The Chlorophyll-chlorophyllido hydrolase 2 gene was significantly up-regulated at 4 days of flooding (Additional file 10: Table S4), which promote the degradation of chlorophyll. Similar results has been reported in rice [44] and mung bean [45]*.* These reductions could be caused by an increase carbohydrate concentration of leaves [24]. Besides, the decrease of chlorophyll and carotenoids is conducive to the photosynthetic structures of plants reduce the absorption of sunlight and prevent photooxidation. In addition, the ratio of chlorophyll a to chlorophyll b were increased. The result showed that plants maintained high photosynthetic ability using adjusting the ratio of chlorophyll a to chlorophyll b in their leaves after being affected of water flooding [45].

On the other hand, when plants are subjected to flooding stress, both light and carbon supplies are limited due to the slower diffusion rates in water, which also reduces plant photosynthesis [43, 46]. Here, great decrease of assimilation rate suggested the vulnerability of photosynthesis in Nakai leaves under flooding. The results consistent with other reported that the decrease of assimilation rate probably due to stomatal closure, chlorophyll degradation and accumulation of reactive oxygen species of leaves. Moreover, transcriptome analysis revealed that many photosynthesis processes were predominantly enriched in KEGG pathways (Additional file 5: Figure S5 and Additional file 10: Table S4**)**. A total 50 DEGs (down-regulated) were detected from the photosynthesis (Ko00195) and photosynthesis - antenna proteins (Ko00196) pathway, which is consistent with a reduction of assimilation rate. Stomata play a pivotal role in photosynthesis by maintaining the balance of gas exchange between the aerial parts of the plant and the atmosphere [47, 48]. With the concomitant decrease in stomatal conductance, flooding caused stomatal limitation on photosynthesis. In the present study, Stomatal conductance and transportation rates have a similar change trend, which agree with the statement that the decline in stomatal conductance would result in reduction of the transpiration under flooding condition. Besides, closure of stomata also should be considered a strategy to adapt the flooding environment [47, 49, 50].

### Physiological response to flooding

Flooding condition lead to hypoxic and anoxic conditions for plants and triggers the accumulation of ROS, which is many causing severe damage to plant cells [10, 15, 44, 47]. Previous studies have showed that many substances and pathways related to ROS scavenging system in plant cells, mainly including enzymatic antioxidants, such as SOD, CAT, POD and APX, and non-enzymatic antioxidants, such as glutathione, ascorbate, tocopherols, and carotenoids. In the present study, the activity of SOD, POD and CAT were significantly increased of Nakai leaves [47]. The result was similar to the previous reports in *Z. mays* [51], *C. indicum* [52], *S. indicum* [53] and *T. aestivum* [21]. Superoxide is converted to O_2_ and H_2_O_2_ using disproportionation reaction induced by SOD. CAT and POD can reduce or eliminate the excess H_2_O_2_ to prevent peroxidation, which can enhance the ability of plants to adapt to flooding stress [54]. Meanwhile, a total 18 DEGs (11 up-regulated and 7 down-regulated) were detected from the peroxisome pathway (Additional file 6: Figure S6). Particularly, the expression of DEGs encoding antioxidant enzymes CAT and POX was significantly higher than the normal conditions, which is consistent with the previous results of the activity of POD and CAT (Fig. 1). Thus, these antioxidant enzymes may be induced by flooding stress and critical for the survival of Nakai leaves in a flooding environment.

However, these antioxidant systems cannot maintain the balance of reactive oxygen systems in plants for a long time. When the oxygen activity system is out of balance, lipid peroxidation is an indicator of oxidative damage under flooding stress. Malondialdehyde (MDA) is a major product of lipid peroxidation, and its increase reflects damage to plant under flooding [55]. The result of significantly increases in the contents of MDA, which indicated that Nakai leaves has excellent resistance to oxidation damage for flooding stress. This result agrees with previous reports [53]. Osmotic regulation is also an important way for plants to reduce osmotic potential and resist stress under flooding stress. Previous studies indicated that soluble sugar and proline are involved in the regulation of osmotic pressure in plant cells, which helps maintain the stability of cells and enhance plants adaptability to flooding stress [24, 25]. Besides, the soluble sugar accumulate also supplies energy for survivor and growth [48, 56, 57]. Here, we observed a marked increase in soluble sugars and proline content after 2 days of flooding, compared to control. The result was similar to the previous reports in Casuarina [58] and wheat [59].

### Hormonal signal transduction and biosynthesis-related DEGs response to flooding

Plant hormones play a crucial role in the process of plant growth and development, as well as in the response to biotic and abiotic stresses [31, 60, 61]. Ethylene is considered to be a pivotal hormone that induce plants to adapt the flooding stress [10, 30]. Such as, the accumulation of ethylene activates the gibberellin pathway to promote the elongation of the rice internode. It’s also can induce the petiole to grow upward, promotes the formation of cellular aerated tissue and the formation of adventitious roots, thus enabling the plant to adapt to the flooding environment [62, 63]. Here, we find 7 DEGs down-regulation in the ethylene synthesis pathway, which different from those of previous studies [60]. But, the result consistent with a study in *Rumex palustris*, which indicated the induction of ethylene can be inhibited under flooding. Besides, ABA is also involved in the process of plant flooding stress [64]. It is produced in mature or aged organism and transported to the younger. The ABA stimulates stomatal closure, which leads to the decreased in stomatal conductance, assimilation rates and transpiration rate of leaves [65]. In this study, the aldehyde oxidase (AAO3) was significantly induced during flooding stress and the ABA 8′-hydroxylase was suppressed, which can promote the accumulation of ABA. Some studies showed that ABA content increased significantly under flooding stress, which can stimulate stomatal closure and increase roots permeability to enhance the water resistance of plants. The result consistent with our study that accumulation of ABA could reduce the stomatal conductance.

In addition to ethylene and ABA associated DEGs response to flooding stress, DEGs associated with the IAA and CK metabolism were also observed. IAA metabolism was observed with 9 DEGs identified during flooding stress, including 6 Aldehyde dehydrogenase, two indole-3-pyruvate monooxygenase (YUCCA) and a BUD13 homolog genes. Present studies suggest that IAA, through ARF gene family, activates stress specific auxin response genes in order to mitigate the negative effects of abiotic stresses [31, 66]. Auxin is the most important endogenous regulator of adventitious root development. It has been confirmed that auxin polarity transport and signal transduction are essential at all stages of root development in *Arabidopsis thaliana*. On the other hand, the specific mechanism of CK action under flooding stress in plants are not fully known. But Zalewski et al. (2010) reported that the productivity of barley is increased by silencing the CK oxidase gene under normal as well as stressed conditions [67]. These results demonstrate that the differential regulation of genes involved in hormone signal transduction and biosynthesis played pivotal role in flooding stress response [11, 68].

### Transcription factors response to flooding

Transcription factors play an important regulator of the response to various biotic or abiotic stresses in plants. Such as drought, flooding, heat or cold and salinity. Some studies reported that these TFs are involved in various abiotic stress and positively improve plants tolerance [69–72]. Thus, it’s have been identified among the most promising targets for the improvement of plant performance under flooding stress. Here, 205 differentially expressed TFs from 31 different families were identified in the Nakai leaves. The most changes were the significant over-representation of MYB and AP2/ERF families in the up-regulated DEGs and down-regulated DEGs in response to flooding, respectively. These TFs DEGs have been reported to mediate abiotic stress responses in plants [71, 72]. Recent studies demonstrated that MYB-superfamily and AP2/ERF genes are modulators of the anaerobic response under low oxygen condition [28, 69, 70]. Such as, the AtMYB44 gene (TRINITY_DN36330_c3_g2) is beneficial for plants to survive by regulating ABA-mediated stomatal closure in response to abiotic stresses and the AtMYB108 gene (TRINITY_DN25829_c2_g5) is also involved in both abiotic stress responses.

### Other metabolic acclimations to flooding stress

Dynamic changes of metabolic pathways in response to submergence, flooding and waterlogging have been reported in a wide range of plants. In general, with the photosynthesis limiting, anaerobic respiration starts to become the primary pathway for supplying energy to the plants. Such as, **s**tarch and sucrose catabolism, glycolysis and ethanoic fermentation to increase substrate-level production of ATP [10, 11, 13]. That is, the plant metabolism from oxidative phosphorylation to anaerobic fermentation to maintain ATP production. Meanwhile, plants are also reduce energy consumption and growth upon flooding conditions [11]. Here, with the GO and KEGG enrichment pathways analysis, many DEGs involved in the starch and sucrose catabolism, glycolysis and pyruvate metabolism pathway were identified. The results supported the notion that flooding promotes anaerobic respiration. Such as pyruvate decarboxylase (PDC) and alcohol dehydrogenase (ADH) are involved in sugar metabolism and are significantly up-regulated during the flooding process. Besides, increase in ADH activity under flooding condition has been reported in many plants [73]. Such as, higher activity of ADH in *Cajanus cajan* genotypes was been reported under waterlogging condition [74]. So, our results also demonstrated that greater ADH activity is contributing to their better survival under flooding condition [75, 76]. Other examples of relevant pathways which are known to be involved in responses to abiotic stresses in general were MAPK signaling pathway, phenylpropanoid biosynthesis and cellulose biosynthetic process, stress response and defense, nitrogen metabolism [69].

## Conclusion

The lack of information available about the molecular mechanisms involved in flooding stress responses in Nakai is currently a major constraint for the further development. In this study, we provided the first characterization of the physiological responses and the transcript profiles of under different levels of flooding treatments in Nakai leaves. Moreover, we examined the accumulation and change of photosynthetic rates, photosynthetic pigments, proline, antioxidative enzyme activity, lipid peroxidation. Besides, many of the unigenes were identified in the present study, which have the potential to be used for the development of novel Nakai varieties with improved productivity and stress tolerance. Thus, the results provide a comprehensive view of the complex molecular events involved in the response to flooding stress of Nakai leaves, which also will promote the research in the development of flood-resistant crops and provide new tools for Nakai breeders.

## Methods

### Plant growth and flooding treatment

*Triarrhena sacchariflora* (Maxim.) Nakai was cultivated in the greenhouse of Wuhan University (These materials are derived from the plant resource garden of Miscanthus in Wuhan University). Plants were cultured with rhizome in pots, each of which contains at least four rhizome of about 10 cm in length. A total 150 potted Nakai plants were cultured in a greenhouse under 400-watt high intensity lamps with a 16 h day and 8 h night photoperiod at a temperature of 23° ± 3 °C. Two months later, we selected 100 potted plants of similar size and divided them equally among two groups. After that two groups of plants were transplanted into same tanks, respectively. The plants in each tank were design consisting of different treatments (normal control and flooding condition) and fore harvest time points: 2-, 4-, 6- and 8-days after flooding (DAF). Leaf from the normal were used as 0-DAF sample and those from the flooding conditions were considered as 2-DAF, 4-DAF, 6-DAF and 8-DAF sample. Each sample was the mixture of leaf from 3 plants and three biological repeats were taken from each period. After each sampling, the sample tissue was immediately frozen using liquid nitrogen and stored at 80 degrees Celsius before use to RNA extraction and other treatments.

### Determination of photosynthetic efficiency-related parameters

In order to understand the difference in photosynthetic indexes between normal and flooding condition plants, the photosynthetic efficiency-related parameters were made by a portable photosynthetic system CIRAS-3(PP SYSTEMS, US). Actinic light was supplied by light-emitting diodes (38% red light, 37% green light, 25% blue light). All measurements were carried out between 09:00 and 10:00 h and carried out six biological duplication. Besides, chlorophyll and carotenoid contents were determined as the method from Wu and Yang [44].

### Determination of antioxidative enzyme activity, proline, lipid peroxidation and soluble sugar

The samples (0.2 g) were used for enzyme extraction. Firstly, we grind the sample with 5 ml sodium phosphate buffer (pH 7.8). Secondly, the sample after grinding was centrifuged at 12,000 g for 20 min. After that, we collect the supernatant will used for determination of enzyme activity. For CAT activity was performed as described previously [43–45]. For SOD activity was determined according to Wu and Yang [44]. For total peroxidase (POD) activity was performed as described previously [45, 46]. Besides, Proline determination was performed as described previously [47, 48]. MDA was quantified using a colorimetric method modified by Wei et al. [53]. Soluble sugar content was performed as described by Mohammadkhani [56]. All experiments carried out three biological duplication.

### RNA sequencing, read assembly, and unigene annotation

Total RNA of six sample were acquired using HiPure Plant RNA Kits (Magen, China). We verified and purified the RNA quality by NanoDrop ND-2000 spectrophotometer (Nano-Drop, Wilmington, DE) and Dynabeads® Oligo (dT) 25 (Life Technologies, USA), respectively. After that, a cDNA library was generated using the purified mRNA, which was used for cluster generation using the TruseqTM RNA sample prep Kit and were subsequently sequenced using the HiSeq 4000 SBS Kit.

To obtain clean reads, the SeqPrep and Sickle software were used to filter the raw data. The clean reads were assembled using Trinity software to generate the unigenes. To identify potential protein-coding genes and annotate the function of these unigenes, all assembled sequences were screened for ORF prediction using EMBOSS software. The predicted protein-coding sequences were searched in the Nr (NCBI non-redundant) database, Pfam database, KEGG (Kyoto Encyclopedia of Genes and Genomes pathway) database, String database, GO (gene ontology) database and Swiss-Prot database using BlastX (E value <1e-5). The proteins with the highest matching value were considered to contain annotation information.

### Identification of differentially expressed genes

In order to test whether the variation of biological duplication between samples accords with the expectation of experimental design, sample correlation and PCA analysis were adopted. Principal component analysis (PCA) and hierarchical clustering were performed to assess transcriptome similarity among samples and to evaluate sampling between biological replicates. To analyze the differential expression of gene/transcript between different samples, the RSEM software was used to calculate the expression of each unigenes. Here, we use the RPKM value to analysis the differential expression between different samples by the edgeR software, filter parameters are as follows: FDR < 0.05 and |log2FC| ≥ 1. Besides, GO enrichment and KEGG enrichment analysis was performed on the differentially expressed unigenes using Goatools and KOBAS software, with Corrected *P*-Value < 0.05.

### Transcription factors

Transcription factors are proteins that bind specifically to DNA sequences and typically has one or more domains that bind to DNA. Transcription factors not only bind to promoter regions in the upstream of genes, but also constitute transcription factor complexes with other transcription factors to affect gene transcription, and thus play an important role in organisms. To get cognate transcription factor information of Nakai, all unigenes were examined from the plant TFDB database by the HMMER analysis method.

### Validation of quantitative real-time PCR (qRT-PCR)

In order to verify the results of transcriptome datas, we selected eight unigenes with significantly different expressions for the expression level verification in the samples by qRT-PCR. We use the primer 6.0 software to design the primers (Additional file 14: Table S8) and madding in AoKe. According to previous results, eIF4a gene was selected as the internal reference gene. The qTR-PCR was executed using the StepOne plusTM Real-Time PCR System. The procedure is as follows: 95 °C for 10 min, 40 cycles at 95 °C for 10 s, 60 °C for 20 s and 72 °C for 20. Each experiment was repeated in three separate biological trials. The results weralyzed using the 2- ΔΔCT method.

### Statistical analysis

One-way analysis of variance (ANOVA) with the least significant differences (LSD, *p* < 0.05) was used to test the flooding effects on the ecophysiological performance of Nakai. Significant differences between control and treatment were determined for physiological and biochemical using SPSS 19.0 (SPSS, Chicago, USA).

## Supplementary information

**Additional file 1: Figure S1.** Samples correlation and PCA analysis

**Additional file 2: Figure S2.** Gene ontology (GO) functional analysis of DEGs between normal and treatment samples.

**Additional file 3: Figure S3.** Heatmap analysis of DEGs related to plant hormone signal transduction. The expressions of gene are displayed different colors. Red means high expression, and blue means low expression.

**Additional file 4: Figure S4.** Heatmap analysis of DEGs related to 205 TFs in different samples. The expressions of gene are displayed different colors. Red means high expression, and blue means low expression.

**Additional file 5: Figure S5.** Heatmap analysis of DEGs related to photosynthesis and photosynthesis - antenna proteins pathway. The expressions of gene are displayed different colors. Red means high expression, and blue means low expression.

**Additional file 6: Figure S6.** Heatmap analysis of DEGs related to peroxisome pathway. The expressions of gene are displayed different colors. Red means high expression, and blue means low expression.

**Additional file 7: Table S1.** The summary of tag numbers

**Additional file 8: Table S2.** The unigenes annotation in different public protein database

**Additional file 9: Table S3.** The unigenes expression statistics

**Additional file 10: Table S4.** The differentially expressed genes (DEGs) in response to flooding

**Additional file 11: Table S5.** Gene ontology (GO) functional enrichment analysis of DEGs between normal and treatment samples.

**Additional file 12: Table S6.** KEGG pathways enrichment analysis of DEGs between normal and treatment samples.

**Additional file 13: Table S7.** Transcription factors response to flooding

**Additional file 14: Table S8.** Primer sequences used in the qRP-PCR experiment

## Data Availability

All data are deposited in supporting information and the datasets generated during the current study are available in the NCBI (SRA accession: SRP158951; https://www.ncbi.nlm.nih.gov/sra/SRP158951).

## Abbreviations

ABA: Abscisic acid
ADH: Alcohol dehydrogenase
CAT: Catalase
CK: Cytokinine
DAF: Days after flooding
DEGs: Differentially expression levels of genes
GA: Gibberellin
GO: Gene ontology
IAA: Auxin
JA: Salicylic acid
KEGG: Kyoto Encyclopedia of Genes and Genomes pathway
LOES: Low-O2 escape syndrome
LOQS: Low-O2 quiescence syndrome
MDA: Malondialdehyde
Nr: NCBI non-redundant
PCA: Principal component analysis
PDC: Pyruvate decarboxylase
POD: Peroxidase
qRT-PCR: Quantitative real-time PCR
ROS: Reactive oxygen species
SOD: Superoxide dismutase
