# Supplementary figures and images for: Transcriptome, physiological and biochemical analysis of Triarrhena sacchariflora in response to flooding stress
Source: BMC Genet. 2019 Nov 29;20:88. doi: 10.1186/s12863-019-0790-4 (PMC6884903; doi:10.1186/s12863-019-0790-4)

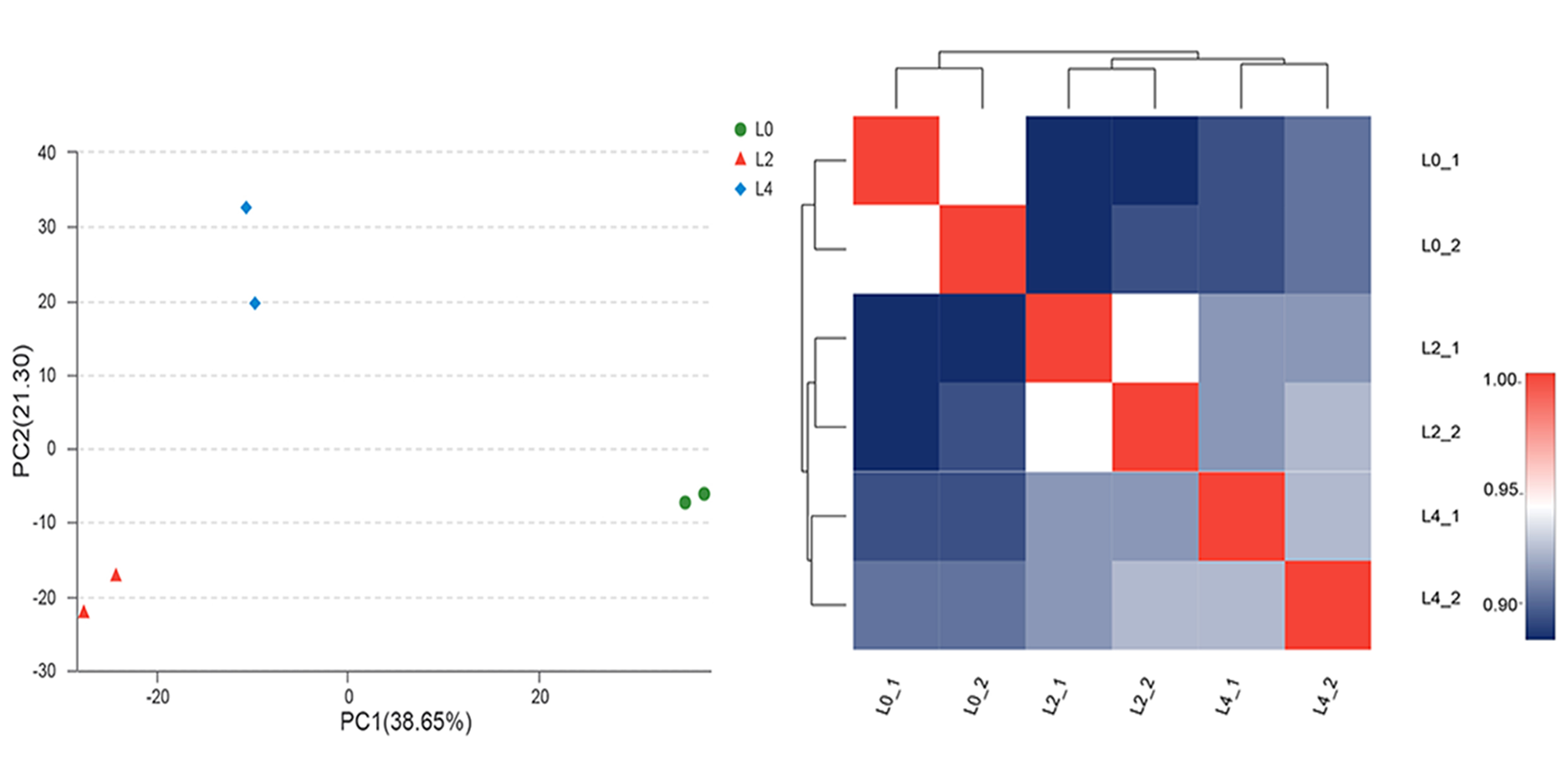

Supplement: Supplementary file 1 — Additional file 1: Figure S1. Samples correlation and PCA analysis [file 12863_2019_790_MOESM1_ESM.jpg]

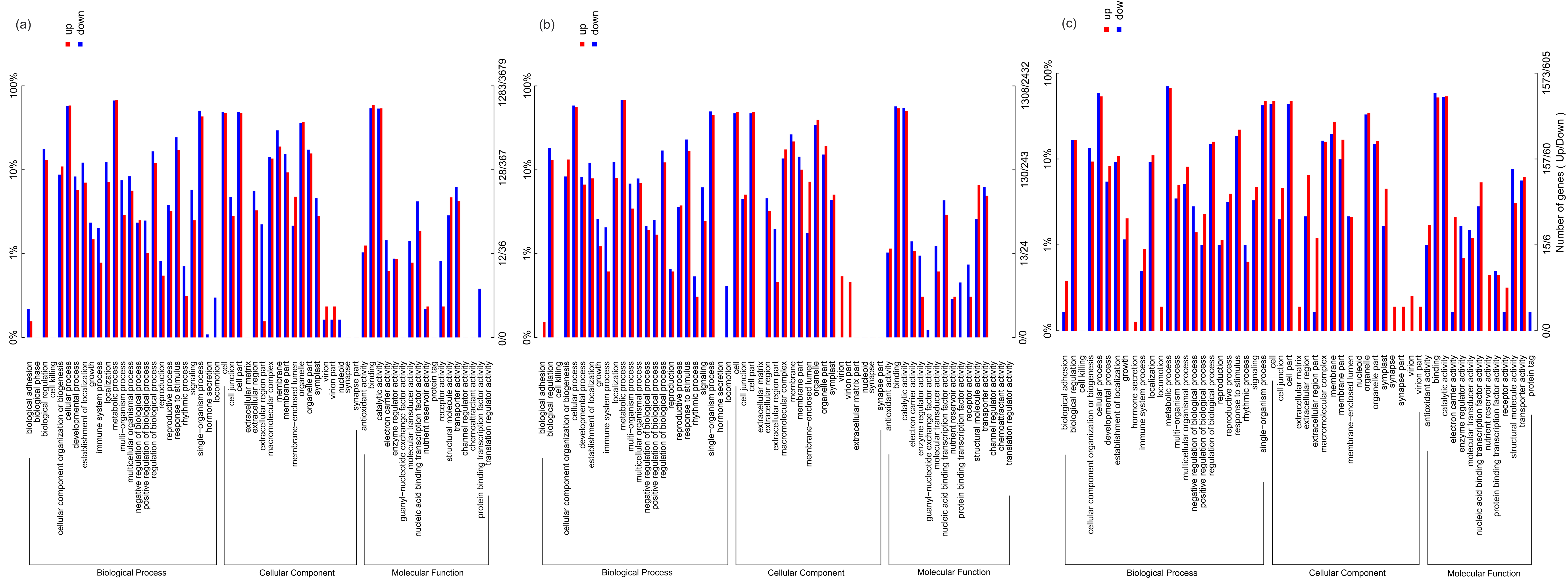

Supplement: Supplementary file 2 — Additional file 2: Figure S2. Gene ontology (GO) functional analysis of DEGs between normal and treatment samples. [file 12863_2019_790_MOESM2_ESM.tif]

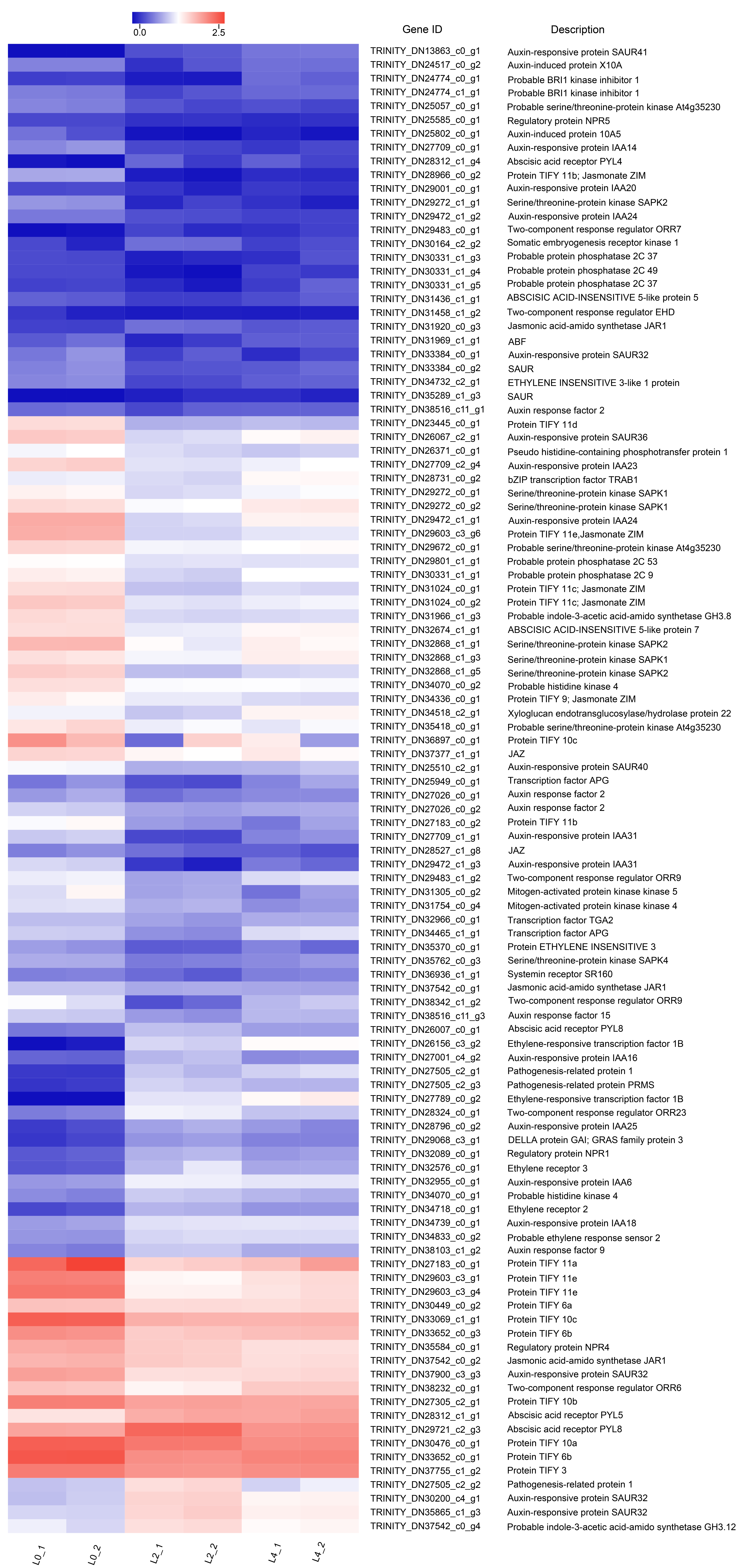

Supplement: Supplementary file 3 — Additional file 3: Figure S3. Heatmap analysis of DEGs related to plant hormone signal transduction. The expressions of gene are displayed different colors. Red means high expression, and blue means low expression. [file 12863_2019_790_MOESM3_ESM.tif]

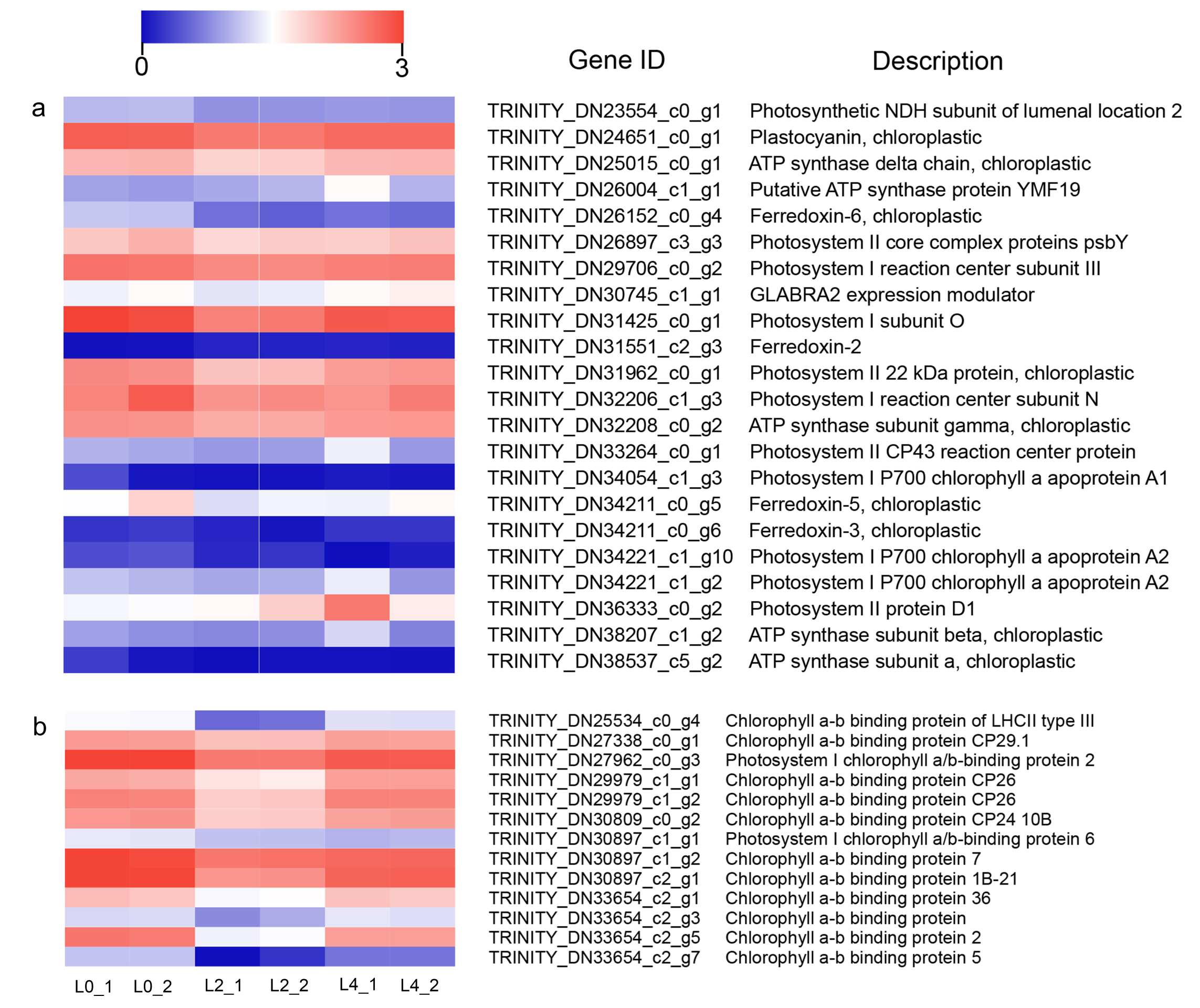

Supplement: Supplementary file 5 — Additional file 5: Figure S5. Heatmap analysis of DEGs related to photosynthesis and photosynthesis - antenna proteins pathway. The expressions of gene are displayed different colors. Red means high expression, and blue means low expression. [file 12863_2019_790_MOESM5_ESM.tif]

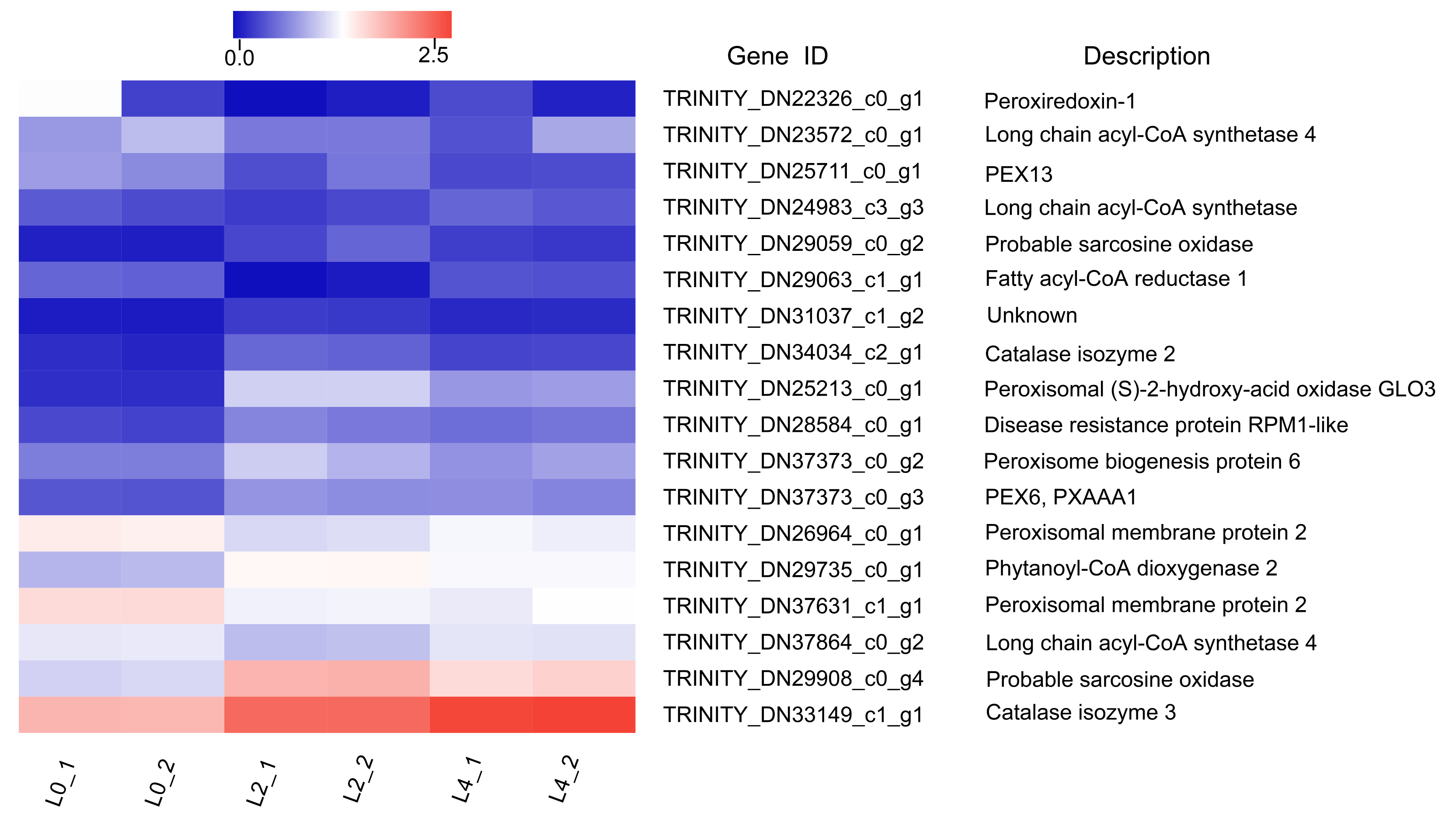

Supplement: Supplementary file 6 — Additional file 6: Figure S6. Heatmap analysis of DEGs related to peroxisome pathway. The expressions of gene are displayed different colors. Red means high expression, and blue means low expression. [file 12863_2019_790_MOESM6_ESM.tif]
